# Cost effectiveness of using surgery versus skeletal traction in management of femoral shaft fractures at Thika level 5 hospital, Kenya

**DOI:** 10.11604/pamj.2013.15.42.2451

**Published:** 2013-06-06

**Authors:** Everisto Opondo, Peter Wanzala, Ansellimo Makokha

**Affiliations:** 1 Jomo Kenyatta University of Agriculture and Technology, College of Health Sciences, Nairobi, Kenya; 2 Kenya Medical Research Institute, Nairobi, Kenya; 3 Jomo Kenyatta University of Agriculture and Technology, Nairobi, Kenya

**Keywords:** Fracture shaft femur, cost effectiveness, functional outcome

## Abstract

**Introduction:**

A prospective quasi experimental study was undertaken at the Thika level 5 hospital. The study aimed to compare the costs of managing femoral shaft fracture by surgery as compared to skeletal traction.

**Methods:**

Sixty nine (46.6%) patients were enrolled in group A and managed surgically by intramedullary nailing while 79 (53.4%) patients were enrolled in group B and managed by skeletal traction. Exclusion criteria included patients with pathological fractures and previous femoral fractures. Data was collected by evaluation of patients in patient bills using a standardized questionnaire. The questionnaire included cost of haematological and radiological tests, bed fees, theatre fees and physiotherapy costs. The data was compiled and analyzed using SPSS version 16. Person's chi square and odds ratios were used to measure associations and risk analysis respectively.

**Results:**

A higher proportion of patients (88.4%) in group A were hospitalized for less than one month compared to 20 patients (30.4%) in group B (p, 0.001).Total cost of treatment in group A was significantly lower than in group B. Nineteen (27.9%) patients who underwent surgery paid a total bill of Ksh 5000-7500 compared to 7(10.4%) who were treated by traction. The financial cost benefit of surgery was further complimented by better functional outcomes.

**Conclusion:**

The data indicates a cost advantage of managing femoral shaft fracture by surgery compared to traction. Furthermore the longer hospital stay in the traction group is associated with more malunion, limb deformity and shortening.

## Introduction

Globally there is a growing incidence of traumatic injury to long bones especially involving the lower limbs. The femur is the largest bone of the body and principal weight bearing bone of the lower extremity that is commonly injured following lower extremity trauma. Femoral fractures are usually associated with considerable mortality and morbidity especially if they follow road traffic accidents. The economic burden of these injuries is felt most in the low resource setting countries with low Gross Domestic Product (GDP) and per capita incomes [1–3].

Over 3,000 Kenyans die as a result of trauma related injuries on Kenyan roads every year, most of them being aged between the ages of 15 and 44 years [3]. The cost to the economy from these accidents has been estimated to exceed US$ 50 million exclusive of the actual loss of life [4].

Unfortunately, road safety trends in Kenya are worsening with more than 75% of road traffic casualties being economically productive young adults in urban areas. Pedestrians and passengers are the most vulnerable and they account for 80% of the deaths from injuries sustained following road traffic accidents. Pedestrians are more likely to be killed in urban areas, whereas passengers are the majority killed on intercity highways that transverse rural setting.

Traction has been the traditional method of treatment of femoral fractures but this method has proved to have a high rate of malunion and knee stiffness. There has been a gradual shift towards surgery as the ideal treatment but very few studies have evaluated the cost effectiveness of the newer interventions in comparison to traction [1].

The goal of treatment is usually restoration of limb anatomy and early mobilization without long term complications [4, 5]. Traditionally in third world countries femoral shaft fractures are managed conservatively by traction and to a small extent by surgery using various nailing and plating techniques. In poor resource setting lack of human capital and material resources may preclude internal fixation of long bone fractures. In early 2000s, the Surgical Implant Generation Network (SIGN) nailing system was introduced in selected district and regional hospitals in Kenya including Thika Level 5 Hospital. It is a solid nail designed for insertion without image intensification, fracture table or use of power reamer and thus specifically adapted for resource poor environments.

Economic cost evaluations (CEA) are widely and commonly used tools in the developed countries for comparison of different interventions and strategies. The purpose of CEA is to assess the relative cost of one intervention over another for a given condition. One intervention is more cost effective if: (1) it is less costly with equal or better outcome or (2) it is more costly with better outcomes, and the added benefit is worth the added cost [3, 4].

This study was carried out to compare the cost effectiveness of managing femoral shaft fractures surgically versus traction at a regional referral hospital in Kenya. The study findings indicate a better cost advantage of treating femoral shaft fractures by intramedullary nailing as compared to using skeletal traction.

## Methods

This prospective quasi-experimental study was carried out at Thika level 5 Hospital located in central province of Kenya from June 2010 to June 2011. Patients were consecutively recruited at casualty and allocated to either of the treatment groups based on treatment modality. Sixty nine (46.6%) patients were enrolled in group A and managed surgically by intramedullary nailing while 79 (53.4%) patients were enrolled in group B and managed by skeletal traction. Among the 148 patients 115(77.7%) were males and 33(22.3%) were females. Age range was 19 to 96 years with a mean age of 42 years. Right femoral fracture was seen in 120(81.1%) patients and left femur fracture in 28(18.9%).

The allocation into each treatment group was done after the patients were given sufficient information on the treatment options and allowed to make a choice. At casualty detailed history about injury, co morbidity and clinical findings were recorded in a questionnaire. Patients diagnosed to have pathological fractures based on history and radiological findings were excluded from the study. After basic data was collected and investigations done the patients in group A underwent surgery while those in group B were managed by skeletal traction (Perkins method). For the patients in group A, intramedullary fixation of the fracture was done on the next elective surgical list. The surgery was done through a lateral approach and the fracture fixed after reduction with an interlocking nail without fluoroscopy. For the patients enrolled into group B skeletal traction was applied within 24 hour from admission with a Steinman's traction pin inserted in the proximal tibia. While in the ward and after discharge all the patients were followed up regularly every two weeks and evaluated for clinical and radiological union and complications.

Review after discharge was done every two weeks for first two months then monthly thereafter. The review in the outpatient clinics included evaluation for fracture healing, local complications and limb mobility. The items considered in treatment cost analysis included the costs of ward bed, drugs, radiographs, laboratory investigations, physiotherapy and theatre fees. The fixed costs such as infrastructure and depreciation value of the initial equipment costs were not considered.

The frequencies and crosstabs procedure were used to create two way and multiway tables. Statistics and graphical displays were used for describing variables, charts and graphs. After tabulation p values were determined using persons’ chi-squares. P value of less than 0.05 was considered significant. All the statistical methods were carried out through the SPSS for windows version 17.0. The results were analyzed for risk of occurrence and associations using the odds ratio and logistic regression.

## Results

### Socio demographics of study population

A total of 148 cases of femoral shaft fractures were seen in the study. 102(75.7%) of the patients were aged between 18 and 50 years with a mean age of 42 years. Males (115) had a significantly higher frequency compared to females (33) (Table 1) with a ratio of 3.5:1. The right side (81.1%) was affected more commonly than the left side (18.9%) (Table 1).

**Table 1:** Distribution of age in years, duration of hospital stay in months and total bill paid in Kenya shillings among the study patients by total and by treatment option

	All patients				
**Variables**	**N**	**Mean**	**SD**	**Minimum**	**Maximum**
Age in years	148	42	19	18	96
Duration of hospital stay in months	148	2	1	1	4
Total bill paid	135	11663	6111	400	40000
**Patients treated with surgery option**					
**Variables**	**N**	**Mean**	**SD**	**Minimum**	**Maximum**
Age in years	69	35	13	18	73
Duration of hospital stay in days	69	30	30	30	90
Total bill paid	68	9761	4565	4750	27000
**Patients treated with conservative option**					
**Variables**	**N**	**Mean**	**SD**	**Minimum**	**Maximum**
Age in years	79	47	22	18	96
Duration of hospital stay in days	79	60	30	30	120
Total bill paid	67	13594	6867	400	40000

Severe trauma due to road traffic accidents was the most common injury mode seen in 83.1% of the patients and mild trauma due to falls was seen in 16.9% of the study patients.

The average duration of hospital stay in the surgery groups was thirty days and sixty days in the conservative group. 61(88.4%) of the patients who underwent surgery were discharged within one month of admission while 24(30.4%) in the traction group were discharged in the same duration.

The average cost of treatment for patients who underwent surgery was Ksh 9761 compared to those managed conservatively Ksh 13594. Thirteen patients had their bills waived due to financial and social reasons.

### Injury patterns

Fracture was seen most commonly in the middle third of the diaphysis. The most common fracture pattern was comminuted and oblique (Table 2). 62.8% of the patients had fracture femur plus one associated systemic injury while 22.3% had more than two injuries systemic injuries and 14.9% had more than three systemic injuries. The most common associated injury was head injury in 27(18.2%) patients followed by other musculoskeletal injuries in 20(13.5%). There was no significant difference in local and systemic complications seen in both groups (Table 3).

**Table 2:** Fracture patterns among the study patients by treatment method

Variables	Total (n=148)		Surgery (n=69)		Conservative (n=79)	
	N	%	N	%	N	%
**Plain femur X-ray**						
Right	120	81.1	53	76.8	67	84.8
Left	28	18.9	16	23.2	12	15.2
**Fracture pattern**						
Transverse	34	23.0	14	20.3	20	25.3
Spiral	20	13.5	13	18.8	7	8.9
Oblique	57	38.5	22	31.9	35	44.3
Comminuted	37	25.0	20	29.0	17	

**Table 3:** Local and systemic complications among the study patients by treatment method

Variables	Total (n=148)		Surgery (n=69)		Conservative (n=79)		OR	95% CI		p value
	N	%	n	%	N	%		Lower	Upper	
**Local complications**										
Yes	72	48.6	33	47.8	39	49.4	Reference			
No	76	51.4	36	52.2	40	50.6	1.06	0.56	2.03	0.852
**Systemic complications**										
Yes	45	30.4	26	37.7	19	24.1	Reference			
No	103	69.6	43	62.3	60	75.9	0.52	0.26	1.06	0.072

### Functional outcomes

Functional outcomes in terms of limb mobility, malunion and limb length discrepancies were analyzed after 12 weeks post trauma. Majority of the patients (55.1%) who underwent surgery attained normal mobility without any support compared to 29.1% in the group managed by traction (OR 3.80 and p 0.004). More patients (43.1%) in the conservative group developed malunion compared to the surgery group with 30.4% having angular malunions greater than 10 degrees radiologically. Overall 58 (84.1%) patients in the surgery group archived union without malunion compared to 45(57%) patients in the conservative group (p=0.001).

## Discussion

With the considerable advances having been achieved in the development of orthopaedic implants it's imperative that newer modes of treatment are analysed against the routine conservative options that are commonly used in developing countries. The evidence derived from such studies should then form the informed basis for changes in patient care especially in resource constrained setting like ours. Patients choice of treatment modalities should based on available evidence of advantage of one method over another.

Throughout the world, there are many sites that have reported reduction in variable costs after use of surgery as compared to traction in treatment of femoral shaft fractures [4–10]. The reduction in costs can be attributed to reduced hospital stay, cost of physiotherapy and X-rays. Gosselin and Lovary in a study on a cohort of 53 patients with similar demographic (mean age of 34.2 and 81% male) to ours treated by Perkins traction in Sierra Leone, a West African country reported malunion rates of 9% and shortening of more than 2cm of 6% [12].

In a study evaluating the impact of introduction of interlocking nail at Mulago hospital in Uganda Sekimpe et, al reported a malunion rate of 8% that compares with our malunion rate of 8.7% for the patients who underwent surgery in this study. The limitation of that study though was the lack of a comparison group.

This study found an average duration of hospital stay in the surgery group to be thirty days and sixty days in the conservative group. A higher proportion of patients (88.4%) who underwent surgery were discharged within one month of admission compared to 30.4% of patients who were treated by traction being discharged in the same duration. The reduced hospital stay in the surgery group lead to reduced bed charges, physiotherapy and radiology costs. The average cost of treatment for patients who underwent surgery was Ksh 9761 compared to those managed conservatively Ksh 13594.

A similar retrospective study in Cambodia at provincial trauma hospital reported the cost of treating one patient in traction as corresponding to treatment of three patient's surgically. That study also reported 57% of patients treated by nailing as having full mobility at discharge and only 22% in the traction group [5]. These results compare favourably with our study where 51% of patients in the surgery group had excellent functional outcome and 29% in the traction group.

## Conclusion

It is safe to conclude that in this setting better clinical outcome was attained at a lower cost with surgery making it more cost effective than Perkins traction in management of adult femoral shaft fractures. The results also confirm the safety of intramedullary nailing at a lower level referral Hospital with minimal equipment.
